# Antiseptics in the Mouth

**Published:** 1888-04

**Authors:** John O. Roe

**Affiliations:** Rochester, N.Y.


### SELECTIONS.

### Antiseptics in the Mouth.

BY JOHN O. ROE, M.D., ROCHESTER, N.Y.,

Fellow of the American Laryngological Association, etc., etc.

There is no place in the human body that furnishes conditions so favorable to the growth of micro-organisms as the mouth. Its constant communication with the external atmosphere, its warmth and moisture, and the great diversity of organic substances that pass through it, each leaving more or less debris about the teeth, and in other recesses of the buccal cavity, afford not only a ready access to all forms of germs, but a comfortable habitation and a sufficiently diversified soil for a large variety of organisms to find abundant sustenance.

The number of distinct species of micro-organisms found in the mouth is therefore great, and they vary in different mouths according to the various kinds of substances found there, upon which these organisms subsist. Twenty-five distinct species of bacteria have been, according to Miller, thus far isolated from the secretions of the human mouth. The largest number found in any one mouth at the same time was eleven, "not including the well-known leptothrix buccalis, spirochæte dentium and vibrio buccalis, which no one has as yet succeeded in cultivating."

Of these fungi it was found that seven would dissolve cooked albumen; five would swell it or render it transparent; ten would dissolve fibrin; four would render it transparent or swell it; nine dissolved gluten; three transformed starch, but only one acted with energy; another seems to live upon it, but without transforming it; seven coagulated milk; six would dissolve casein; nine transform lactose into lactic acid; seven intervest cane sugar; seven caused glucose to ferment and transform it partially into alcohol. The action of all of these was more or less energetic.

The above enumerated varieties of organisms are those found in healthy mouths, or in the mouths of persons in an undoubted state of health. In diseases of the mouth or the throat, this number is greatly increased according to the nature of the disease. Thus in diphtheria we find the micrococcus diphthereticus, an organism peculiar to this disease. In stomatitis, bacteria are found peculiar to it; and in erysipelas of the throat a bacterium peculiar to that disease.

It is quite generally believed that in health the organisms found in the mouth are entirely harmless, and that they can continue to grow and multiply from generation to generation without our concern. This belief is not warrantable, for not only do these germs continue to evolve noxious gases to taint the breath, or to convert harmless substances into poisonous compounds, but it has been shown by experimental investigation that they are the active agents in the causation of dental caries. The relation of cause and effect between these organisms and the production of dental caries has been pointed out by Weil, Underwood, Milles, Hueppe, and Miller. Messrs. Underwood and Milles, of London, in a paper read by them conjointly before the dental section of the International Medical Congress held in London in 1881, * presented their conclusions on this subject as follows: "We consider that caries is absolutely dependent upon the presence and proliferation of organisms; that these organisms attack first the organic material, and feeding upon it, create an acid which removes the lime salts; and that all the difference between caries and simple decalcification by acids is due to the presence and operation of germs. This view we propose to call the "septic theory."

In confirmation of these observations, experiments were made by them in the artificial production of caries by introducing into flasks the substances and conditions usually found in the mouth. But in every instance in which the contents of the flasks were rendered aseptic no caries could be produced. These statements have been substantially verified by Dr. W. D. Miller, † formerly of New York, and now of Berlin, Germany. He has shown by a large number of ably and carefully conducted experiments, that

* Transaction of the International Med. Con., London, 1881. Vol. III. p. 523.
† *Vide* the articles by him on this subject that have appeared in different German periodicals and also in the "*Independent Practitioner*" during the past four years.

caries can be produced only by the process of fermentation and decomposition, and these, as we all know, are induced by the presence of micro-organisms.

I will not attempt to go into the details of this subject, for it would extend this article far beyond the intended limit, but I will summarize as briefly as possible the results of Dr. Miller's investigations regarding the characteristics of the twenty-five different species above referred to. Of these ten were micro-ordiplococci, five were bacteria, and six were bacilli, while some showed more than one form of development. In liquid media, three grew out into long leptothrix, while one developed into spirilli. Eight were motile, fourteen non-motile, while three were seen to form spores. The others multiplied by division alone. Eight liquefied gelatine; one converted it into a paste; thirteen left it unchanged. Ten were strictly ærobian; four were not strictly ærobian; eight grew equally well with or without air. Sixteen produced an acid reaction in a solution of beef extract, peptone and sugar. Four produced an alkaline reaction with no bad-smelling products and left the solution neutral. Some produced acid reaction in fermentable solutions and alkaline reaction in non-fermentable solutions. The acid produced in nearly all was lactic acid; other acids were not determined. Thirteen were cultivated on a potato; five of them grew rapidly, one of them particularly so. Of fifteen cultivated on boiled white of egg, four grew rapidly with the evolution of sulphuretted hydrogen and ammonia. Seven grew slowly; four scarcely at all. They converted egg into peptone; and in two cases more than they consumed, leaving free peptone. Some produced in fermentable solutions large quantities of gas, but much less gas in non-fermentable solutions. Four produced coloring matter of brick yellow masses, such as seen occasionally on the buccal surface of teeth that are not kept clean. On potato the same fungi produced a bright yellow coloring matter. Fungi developed rapidly on decalcified dentine, when kept in a damp cell, with destruction to the dentine; but on normal dentine or dentine not decalcified, they would have no effect. From these studies of the behavior of the different forms of bacteria found in the human mouth we can readily understand the *modus operandi* in which they become the agents in causing dental caries. The normal secretions formed in the mouth are alkaline. The abundant flow of the alkaline fluids from the parotid, sub-maxillary, and sub-lingual glands during the taking of foods is unquestionably a provision of nature to keep the teeth surrounded by an alkaline fluid in order to neutralize such acids as are often taken with foods and drinks.

It is therefore between the times of eating, when the glandular secretions are scanty, that an acid can have its most destructive effect, and this is the time when we find that lactic acid is produced most abundantly by this fermentative process. Some traces of butyric acid have been found in the mouth, but no acetic acid except that taken as a condiment.

Acetic acid can not result from fermentation in the mouth, for the reason that acetic acid fermentation cannot go on at a temperature above 35° C. (95° F).

Thus we see that all the acids produced in the mouth must come from hydro-carbon or fermentable substances, and fermenting solutions almost invariably have an acid reaction. The fermentation of solutions containing more or less grape or cane sugar, are, when air is entirely excluded, vinous, as in the manufacture of wines—alcohol and carbonic acid being evolved; but when air is freely admitted the fermentation goes on to the formation of acetic acid if the temperature is below 35° C., but if above 35° C. lactic acid results.

In nitrogenous or non-fermentable solutions decomposition takes place, evolving free ammonia and, if sulphur is present, sulphuretted hydrogen, and the reaction remains neutral or alkaline; but if sugar or a hydro-carbon be added these same bacteria at once institute a fermentative process and in a short time the same solution will become acid from the lactic acid that has been formed by the fermentation.

It is this acid thus generated that attacks the lime salts of the teeth and leaves the organic portion of the teeth exposed to be digested by the fungi.

As has been shown by Underwood, Milles, and Miller, dentine decalcified by acids may be kept for an indefinite period in an aseptic solution, either acid or alkaline; but as soon as bacteria are admitted the decalcified dentine will undergo a process of softening and will in a short time be destroyed. In the article referred to, by Underwood and Milles, they hold that the bacteria attacked first the organic portion and from this generated the acid that decalcified the dentine. They have doubtless since seen, as Miller has shown, that before the bacteria can get at the dentine they must first generate an acid to decalcify it, assisted doubtless somewhat by acids taken as condiments. If this solution is examined a ferment will be found like pepsin, which, if isolated, will dissolve coagulated albumed and also digest decalcified dentine. Thus these energetic little organisms, in order to get at the dentine, to add it to their store of condiments, first institute a fermentation in the carbo-hydrates which they find in the mouth, by which they generate their acid to remove the lime salts, which is the barrier that prevents them from devouring it, and also maufactures a peptone by which they peptonize the dentine in order to prepare it for their food.

We have also seen that many of these bacteria are ærobian, while many of them are not—that is, they grow equally well without air. In this manner they are able to penetrate the dental tubules and to live and propagate under a filling. The necessity is apparent of rendering the prepared tooth cavities thoroughly aseptic, but the fillings and instruments with which they are put in should be thoroughly sterilized.

In the mouth we may have, therefore, a large number of chemical combinations and decompositions going on at the same time, which are varied according to the substances taken into the mouth.

It is not the fact of the presence of micro-organisms that is of any significance, but it is the compounds they generate that are to be feared, as pointed out by Cheyne.* Thus about the teeth, they are as harmless, until they produce the acid to decalcify the dentine, as the small snake is without its venom.

It is undoubtedly in this manner that these micro-organisms perform an active part in such zymotic diseases as diphtheria.

*"Antiseptic Treatment of Wounds." London, 1885, p. 14.

The favorable soil being presented they at once not only multiply rapidly, but go actively to work to produce a poison with which to overcome their victim.

Many experiments have been instituted from time to time to discover if diphtheria could be induced without the presence of bacteria. The membranous exudate from the throat of diphtheric patients was taken, macerated, the solution percolated through porous clay and thoroughly sterilized. With this solution entirely free from all micro-organisms rabbits were inoculated, and in every instance diphtheria was developed or they died from septic poisoning. From this it was concluded that diphtheria had nothing to do with germs, but that the poison was of a chemical nature, it being entirely overlooked that this chemical poison was undoubtedly manufactured by the bacteria.

Cases have been reported of persons dying from the effects of the bite of chickens. We all know that chickens are not poisonous, nor do they live on substances that would poison human beings, but in the mouths of these chickens certain poisonous compounds had been developed by micro-organisms that when introduced into the circulation of these persons resulted in their death in a few hours. Oft-times the bite of other domesticated animals will produce very serious symptoms of septic poisoning. In all such cases a thorough examination, both chemical and microscopic, should be made of the contents of the animal's mouth to determine the specific cause. So it is often the case, as Miller remarks, that a slight scratch on the finger from a root in an unhealthy human mouth may produce the most dangerous symptoms of blood poisoning.

Fermentative, putrefactive and septic conditions in the mouth and the subject of dental caries receive altogether too little attention from the majority of the members of the medical profession. They altogether too frequently leave the mouth and the teeth to the care of the individual and the dentist, and in many instances the latter is not consulted until many of the teeth are irreparably decayed. The teeth of children, and particularly those of the second dentition, as well as those of adults should be frequently looked into by the surgeon, who should see that the mouth is kept in a thoroughly antiseptic condition and that the first evidence of decay receives the proper attention by a competent dentist. The importance of sound teeth cannot be over-estimated when we consider the injurious effect of diseased teeth on surrounding organs, especially on the ears, eyes, throat, and nose. The patient should be made to understand that the object of thoroughly cleansing the teeth is not only for the purpose of making them white and to appear clean on the outer surface, but to remove from every portion of the mouth all substances that can ferment and decompose and to render the teeth as thoroughly as possible aseptic. The use of the brush, quill tooth-pick and floss silk, as advised by dentists, for the removal of the debris of food lodged about the teeth, is important, as is also the frequent employment of antiseptic washes for the destruction of the germs harboring about them.

In the use of antiseptics in the mouth the general principles apply, as in the employment of antiseptics in the treatment of wounds— that is, the prevention of fermentation and decomposition. When it was demonstrated by Pasteur that fermentation and decomposition were induced by germs, it could be seen at once that to prevent these processes we had simply to exclude the germs or destroy those which might be present, or remove altogether the substances on which they subsist. The practical application of this knowledge to the requirements of surgery and the name of Lister is inseparable; for operations now are simplified, and many are performed that were not deemed possible by surgeons before the days of antiseptic surgery. Not only do the principles of antiseptics apply in the treatment of wounds, but they are equally important in the preservation of normal structures from the destructive effects of micro-organisms. The importance of maintaining the mouth in a thoroughly sanitary condition cannot be too strongly emphasized, when we consider the disastrous effects on the teeth of an unsanitary condition of the mouth, as well as the readiness with which diseased germs cultivated in the mouth find access to the stomach, and the extent of the actively absorbing surface which the mouth and fauces afford, for the absorbing of septic compounds directly into the circulation.

